# The quantum chemical causality of pMHC-TCR biological avidity: Peptide atomic coordination data and the electronic state of agonist N termini

**DOI:** 10.1016/j.dib.2015.02.021

**Published:** 2015-03-20

**Authors:** Georgios S.E. Antipas, Anastasios E. Germenis

**Affiliations:** a Division of Materials Technology, National Technical University of Athens, Zografou Campus, Athens 15780, Greece; b Department of Immunology & Histocompatibility, School of Medicine, University of Thessaly, Biopolis, Larissa 41110, Greece

**Keywords:** Immunology, Biochemistry, Materials Science, Quantum Chemistry, Class I MHC, CD8+, Cytotoxic Lymphocytes, Protein–protein interactions

## Abstract

The quantum state of functional avidity of the synapse formed between a peptide-Major Histocompatibility Complex (pMHC) and a T cell receptor (TCR) is a subject not previously touched upon. Here we present atomic pair correlation meta-data based on crystalized tertiary structures of the Tax (HTLV-1) peptide along with three artificially altered variants, all of which were presented by the (Class I) HLA-A201 protein in complexation with the human (CD8^+^) A6TCR. The meta-data reveal the existence of a direct relationship between pMHC-TCR functional avidity (agonist/antagonist) and peptide pair distribution function (PDF). In this context, antagonist peptides are consistently under-coordinated in respect to Tax. Moreover, Density Functional Theory (DFT) datasets in the BLYP/TZ2P level of theory resulting from relaxation of the H species on peptide tertiary structures reveal that the coordination requirement of agonist peptides is also expressed as a physical observable of the protonation state of their N termini: agonistic peptides are always found to retain a stable ammonium (NH_3_^+^) terminal group while antagonist peptides are not.

## Specifications table

**Table:** 

Subject area	*Immunology, Biochemistry, Materials Science, Quantum Chemistry*
More specific subject area	*Class I MHC, CD8+ Cytotoxic Lymphocytes, Protein–protein interactions*
Type of data	*Excel spreadsheet*
How data was acquired	*Data from crystallized tertiary structures was acquired from the Protein Data Bank (PDB)*
Data format	*Text*
Experimental factors	*None*
Experimental features	*None*
Data source location	*Not applicable*
Data accessibility	*Data is with this article*

## Value of the data

• Cumulative over-coordination (higher density) in respect to the native peptide (Tax) signifies a weaker agonist peptide, while cumulative under-coordination (lower density) flags an antagonist peptide.
• Pronounced coordination deviations from the native peptide signify correspondingly weaker peptide avidity, as portrayed by the behavior of the weak agonist (V7R) and the weak antagonist (Y8A). Therefore, cumulative coordination difference in respect to the native peptide is a physical observable symptomatic of biological function, albeit on the provision that the stereochemistry of the native peptide is known.
• The protonation state of the peptide N-terminus is a physical observable linked to peptide functionality, which may be employed to characterize avidity even when native peptide stereochemistry is unavailable. Furthermore, if the N terminus of agonist peptides were to be found to retain its protonation (ammonium) state during the engagement it would signify the presence of causality.

## 1 Experimental design, materials and methods

### 1.1 Peptides

The current study has considered a set of crystallized pMHC-TCR complexes which appear stereochemically similar but have been functionally characterized as very diverse [1]. All peptides are presented by HLA-A201 and bound to the human A6TCR. These were the cognate HTLV-1 Tax peptide (LLFGYPVYV, PDB accession code 1AO7) (index peptide) and three variants which were produced by artificial substitution of single residues along the primary Tax structure: V7R (LLFGYPRYV, PDB accession code 1QSE), Y8A (LLFGYPVAV, PDB accession code 1QSF) and P6A (LLFGYAVYV, PDB accession code 1QRN). Based on measurements of functional avidity, P6A and Y8A behave as a strong and a weak antagonist respectively, while V7R behaves as a weak agonist or null peptide [1]. Additionally, the variant P6EtG (LLFGYEtGVYV), characterized as a super agonist by kinetic and thermodynamic measurements [2], was reconstructed in-silico from the protonated P6A tertiary structure via substitution of the A6 by an EtG residue and subsequent DFT relaxation of the EtG atoms along with peptide atoms within a radius of 4 Å of each of the EtG atom centers; P6EtG was subsequently compared against the index peptide.

**Fig. 1:**
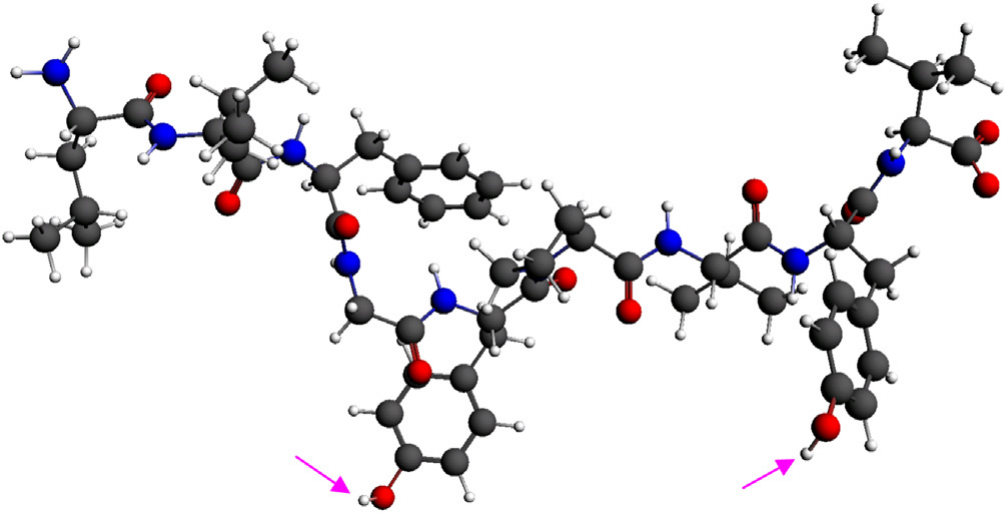
Protonated Tax tertiary structure. Atom color notation is C – gray, N – blue and O – red. The figure serves as an example depicting the protonation/deprotonation possibilities of the (hydrophilic) hydroxyl groups attached to phenol side chains in positions 5 and 8 for all peptide models listed in Table 1.

All PDB tertiary structures represent unprotonated complexes which are crystallographed via X ray diffraction (the H species are weak scatterers and may not be recorded by XRD). Hence, in the current study, all structures were saturated with protons and the H species was subsequently relaxed via DFT while keeping backbone and side-chain atoms frozen in their original (crystallographed) positions. Moreover, the effect of spin/charge combinations on the electronic structure of the peptides was studied in the gas phase; this decision was made on the basis that fully formed pMHC complexes in engagement with the TCR tend to be associated with a high entropic benefit for the efficient expulsion of water molecules from the immune synapse [3]. Nevertheless, whether or not water molecules are, in fact, retained within the synapse, they may only influence the protonation state of terminal groups of the hydrophilic part of the peptide (i.e. side chains in positions 5 and 8, also see atoms marked by cyan arrows in Fig. 1). This eventuality has been taken into account by considering various protonation possibilities (see Table 1).

### 1.2 Calculation of pair correlation functions

The PDF, also referred to as *g*(*r*), is a statistical representation of interatomic distances [4]. The PDF was calculated by initially constructing the histogram of interatomic distances in respect to the real space coordinate, *r*. Calculation of the histogram involved the initial partition of space into bins of finite width. The most suitable bin size, Δ*r*, is a matter of experimentation and it is desirable to select the largest bin size for which fine PDF details are maintained. Here, the bin size was set to 0.1 Å, after a number of trials. The PDF is given by the expression

(1) $$g(r)=\frac{1}{2\pi Nr^{2}\rho_{0}}\sum_{j=1}^{N}\sum_{i>j}^{N}\delta (r-r_{ij})$$

where *N* is the number of peptide atoms, *δ* is the Dirac delta function, *r*_*ij*_ represents the interatomic distance between atoms *i* and *j* and *ρ*_0_ is the number density expressed as *N*/*V*, where *V* is the volume of the simulation box containing the peptide. Based on the PDF, the expression of the radial distribution function (RDF), also referred to as *R*(*r*), may then be defined as

(2) $$R\left(r\right)=4\pi r^{2}\rho_{0}g\left(r\right)$$

Eq. (2) is utilized in the calculation of atomic coordination, the latter defined as the number of atoms,$n_{r_{1}}^{r_{2}}$, within a spherical shell restricted by radii *r*_1_ and *r*_2_, where *r*_1_<*r*_2_, along the real space coordinate

(3) $$n_{r_{1}}^{r_{2}}=\int_{r_{1}}^{r_{2}}R(r)dr=4\pi \rho_{0}\int_{r_{1}}^{r_{2}}g(r)r^{2}dr$$

Eq. (3) yields the average coordination between *r*_1_ and *r*_2_. The cumulative coordination for each peptide up to any value of interatomic distance *r*_2_ may then be defined as the integral of (3) by setting *r*_1_ equal to zero. Additionally, the running difference between any pair of such cumulative coordination integrals may also be calculated. Coordination as defined by (3) is independent of atomic species and its pair distribution function is, therefore, referred to as the total PDF. If the interatomic distance, *r*_*ij*_, is calculated for specific pairs of atomic species (partials), Eq. (3) then yields the atomic partial coordination, for which cumulative coordination differences may also be calculated.

In the current work, the very important limit of the first coordination shell of peptide tertiary structure was also estimated via the PDF. The first coordination shell comprises the first peak of the total PDF and it is made up by all bonded interactions, regardless of their respective placement within the structure.

### 1.3 Quantum chemical calculations

DFT open-shell, all-electron calculations were performed with the Amsterdam density functional (ADF) program [5–8]. Electron exchange and correlation was addressed by the BLYP [9,10] functional in the generalized gradient approximation (GGA) scheme. Atomic orbitals were Slater-type, expressed as single-electron wavefunctions, expanded by a triple-ζ basis set with two sets of polarization functions (TZ2P). Relaxation simulations were followed by single point calculations for all structures to ensure full SCF convergence; Simulations which yielded non-aufbau electron occupations were discarded.

## Figures and Tables

**Table 1:** Peptide models considered. Model notation is: peptide name followed ‘*z*’ and ‘*s*’ denoting peptide formal charge and spin polarization, respectively. In every model, protonated hydrophilic side chains are denoted by ‘*p*’ while deprotonated side chains are symbolized by ‘*u*’.

**Model designation**	Tax z0 s0 p	Tax z-2 s0 u	Tax z-2 s2 u
**Peptide**	Tax	Tax	Tax
**Immunological designation**	Agonist		
**Sequence** (contributors to z in bold, variants in respect to Tax underlined)	**L**LFGYPVY**V**		
**Formal charge**	0	−2	−2
**Spin polarization**	0	0	2


**Model designation**	V7R z-1 s1 u	V7R z1 s0 p
**Peptide**	V7R	V7R
**Immunological designation**	Weak agonist	
**Sequence** (contributors to z in bold, variants in respect to Tax underlined)	**L**LFG**Y**PR**YV**	
**Formal charge**	−1	1
**Spin polarization**	1	0


**Model designation**	Y8A z-1 s0 u	Y8A z0 s0 p
**Peptide**	Y8A	Y8A
**Immunological designation**	Weak antagonist	Weak antagonist
**Sequence** (contributors to z in bold, variants in respect to Tax underlined)	**L**LFG**Y**PVA**V**	**L**LFGYPVA**V**
**Formal charge**	−1	0
**Spin polarization**	0	0


**Model designation**	P6EtG z0 s2 p	P6EtG z-2 s0 u	P6EtG z-2 s2 u
**Peptide**	P6EtG	P6EtG	P6EtG
**Immunological designation**	Super agonist		
**Sequence** (contributors to z in bold, variants in respect to Tax underlined)	**L**LFGY EtG VY**V**		
**Formal charge**	0	−2	−2
**Spin polarization**	2	0	2


**Model designation**	P6A z0 s0 p	P6A z-2 s0 u	P6A z-2 s2 u
**Peptide**	P6A	P6A	
**Immunological designation**	Antagonist		
**Sequence** (contributors to z in bold, variants in respect to Tax underlined)	**L**LFGYAVY**V**		
**Formal charge**	0	−2	−2
**Spin polarization**	0	0	2
